# Differential blood miRNA expression in brain amyloid imaging-defined Alzheimer’s disease and controls

**DOI:** 10.1186/s13195-020-00627-0

**Published:** 2020-05-15

**Authors:** Helen Zong Ying Wu, Anbupalam Thalamuthu, Lesley Cheng, Christopher Fowler, Colin L. Masters, Perminder Sachdev, Karen A. Mather, and the Australian Imaging Biomarkers and Lifestyle Flagship Study of Ageing

**Affiliations:** 1 Centre for Healthy Brain and Ageing, University of New South Wales, Sydney, Australia; 2 Neuroscience Research Australia, Sydney, Australia; 3 Department of Biochemistry and Genetics, La Trobe Institute for Molecular Science, La Trobe University, Bundoora, Victoria Australia; 4 Florey Institute, University of Melbourne, Melbourne, Australia; 5 Neuropsychiatric Institute, Euroa Centre, Prince of Wales Hospital, Sydney, Australia

**Keywords:** Alzheimer’s disease, Biomarker, MicroRNA, Amyloid imaging, Early diagnosis

## Abstract

**Background:**

Peripheral blood microRNAs (miRNA) have been identified as potential biomarkers for Alzheimer’s disease (AD). Study results have generally been inconsistent and limited by sample heterogeneity. The aim of this study is to establish candidate blood miRNA biomarkers for AD by comparing differences in miRNA expression between participants with brain amyloid imaging-defined AD and normal cognition.

**Methods:**

Blood RNA was extracted from a subset of participants from the Australian Imaging Biomarkers Lifestyle Study of Ageing cohort (AIBL) with brain amyloid imaging results. MiRNA profiling was performed using small RNA sequencing on 71 participants, comprising 40 AD with high brain amyloid burden on imaging (amyloid positive) and 31 cognitively normal controls with low brain amyloid burden (amyloid negative). Cross-sectional comparisons were made between groups to examine differential miRNA expression levels using Fisher’s exact tests. Replication of results was undertaken using a publicly available dataset of blood miRNA data of AD and controls. In silico analysis of downstream messenger RNA targets of candidate miRNAs was performed to elucidate potential biological function.

**Results:**

After quality control, 816 miRNAs were available for analysis. There were 71 significantly differentially expressed miRNAs between the AD and control groups (*p* < 0.05). Two of these miRNAs, miR-146b-5p and miR-15b-5p, were also significant in the replication cohort. Pathways analysis showed these miRNAs to be involved in innate immune system and regulation of the cell cycle, respectively, both of which have relevance to AD pathogenesis.

**Conclusion:**

Blood miR-146b-5p and miR15b-5p showed consistent differential expression in AD compared to controls. Further replication and translational studies in strictly phenotyped cohorts are needed to establish their role as biomarkers for AD to have clinical utility.

## Background

AD is a neurodegenerative disease characterised by deposition of amyloid β (Aβ) in neuritic plaques extracellularly and the intracellular formation of neurofibrillary tangles of hyperphosphorylated tau protein. It is a major source of disease burden worldwide; however, currently, there are no effective treatments. It is well recognised that AD is a biological and clinical continuum. It begins at a preclinical stage and, as molecular alterations and neurodegeneration accumulate, progresses through to mild cognitive impairment due to AD (prodromal AD), towards mild, moderate, and severe dementia stages [1]. The National Institute on Aging and Alzheimer’s Association (NIA-AA) research framework defines AD biologically, by neuropathologic change or biomarkers [2]. Biomarkers are grouped into those of β amyloid deposition (e.g. through cerebrospinal fluid (CSF) measurements or positron emission tomography (PET) imaging), pathologic tau, and neurodegeneration [AT(N)]. The relative high cost of PET and invasive nature of CSF sampling limit the accessibility and feasibility of these biomarkers for routine clinical use. Blood-based biomarkers would offer great advantage as they are easily accessible and well tolerated in the clinical setting. Current blood biomarkers are limited and there is a need for the discovery of new blood-based biomarkers to enable early and accurate diagnosis of the disease. Alternatively, these less-invasive and less-expensive blood-based biomarkers may play a screening role in selecting individuals for more expensive/invasive testing [2]. Furthermore, amyloid and tau may explain only some aspects of AD pathophysiology; the discovery of new biomarkers could potentially lead to new insights into AD biology and therapeutic targets.

MicroRNAs (miRNAs) have been recognised as novel biomarkers of diseases because of their diverse tissue- and cell-specific biological and pathological functions [3]. They are a class of short non-coding RNA, of approximately 22 nucleotides in length, which in general post-transcriptionally downregulate protein expression. MiRNAs bind to complementary sites located in the three prime untranslated regions (3′ UTRs) of their target messenger RNAs (mRNAs) resulting in inhibition of translation [4, 5]. Their dysregulation has been implicated in various pathological conditions. Prior studies have proposed candidate miRNA biomarkers for AD, with differential expression of miRNAs in AD compared to control groups [6–8]; however, results of these biomarker studies have been inconsistent with a lack of reproducibility and validation of candidate miRNAs across studies. One constraint is the phenotypic variability across cohorts [6]. Most of the current studies define their cohorts based on clinical criteria. There is significant discordance between the clinical diagnosis of AD with post-mortem examination for AD pathology [9]. Furthermore, individuals without cognitive impairment in the control cohort may harbour amyloid pathology at post-mortem. When searching for new biomarkers, it is important that cohort phenotypes are strictly defined, and the addition of brain amyloid imaging using positron emission tomography (PET) to identify Aβ burden in life improves the robustness of the diagnostic groups, especially when examining pre-clinical or early AD [2].

In this study, peripheral blood miRNA expression was examined in a well-phenotyped cohort with assessment of brain amyloid burden to support the clinical diagnosis of AD and a non-AD comparison group. Using small-RNA sequencing, differential miRNA expression among participants with amyloid-positive AD compared to amyloid-negative cognitively normal controls was assessed. Replication was undertaken in an independent cohort. The potential biological functions of candidate miRNAs and role in AD pathology were assessed using in silico analyses of mRNA targets and pathways.

## Methods

### Sample selection

This study is a cross-sectional analysis of a subset of participants from the Australian Imaging Biomarkers Lifestyle Study of Ageing (AIBL) cohort. The methodology of recruitment for the AIBL study has been previously described [10]. AIBL is a longitudinal study involving participants with AD, mild cognitive impairment, and healthy controls. A subset of the AIBL cohort underwent amyloid PET imaging at baseline and at 18-monthly intervals to measure brain Aβ burden [11]. Four different Aβ tracer compounds have been used in the AIBL cohort and participants were characterised as amyloid-positive or amyloid-negative based on tracer-specific standardised uptake value ratio (SUVR) defined by the AIBL research group using their CSIRO-developed CapAIBL PET quantification algorithm. The thresholds used were PiB (SUVR 1.4), NAV4694 (SUVR 1.4), Flutemetamol (SUVR 0.55), and Florbetapir (SUVR 1.05) [12]. The thresholds were selected as per their association with risk of disease progression.

From the subset of the AIBL cohort with amyloid scans, participants were selected for our study if they met the following criteria:

1. PAXgene tubes of whole blood available for RNA extraction AND
2. Amyloid imaging data indicating high Aβ burden (amyloid positive) for the AD group OR
3. Amyloid imaging data indicating low Aβ burden (amyloid negative) for the non-AD cognitively normal control group

Additional criteria for the amyloid negative control group include the following:

1. A minimum of 36 months of cognitively normal diagnosis AND
2. A minimum of two amyloid negative scans (36 months follow-up) on PET imaging

Those who converted to MCI or AD during the follow-up period of up to 108 months were excluded.

The institutional ethics committees of Austin Health, St. Vincent’s Health, Hollywood Private Hospital, and Edith Cowan University granted ethics approval for the AIBL study. All volunteers gave written informed consent before participating in the study.

### RNA extraction

Total RNA including miRNA was isolated using the PAXgene Blood miRNA Kit (Qiagen, Germany) following the manufacturer’s recommendations. RNA integrity was analysed using Bioanalyzer 2100 (Agilent, USA), and concentration and purity of total RNA (including miRNA) were quantified using the NanoDrop 2000 UV-spectrophotometer (Thermo Scientific, Wilmington, DE, USA). RNA samples were concentrated by Speedy vacuum to standardise samples to 60 ng/ul followed by quality checks using the Xpose (Trinean) and TapeStation (Agilent). The amount of RNA used for the sequencing assay was 5 μl (i.e. 300 ng).

### High-throughput expression profiling of miRNAs

Library preparation and sequencing was performed by the Ramaciotti Centre for Genomics, University of New South Wales. Small RNA samples were converted to Illumina sequencing libraries using the QIAseq Small RNA-seq prep (Qiagen, Germany), following the manufacturer’s protocol. Libraries were normalised to 6 nM and then pooled adding 26 libraries per pool. Each pool was sequenced using one NextSeq 500 1 × 75bp High Output flowcell and NextSeq 500/550 v2 kits, generating 75 bp single-end reads, with approximately 10 million reads per sample. A pass threshold of > 85% of bases higher than Q30 was used. FASTQ files were generated using bcl2fastq2.

The sRNAnalyzer pipeline [13] was used for pre-processing, alignment, and summarising the read counts of the miRNA sequenced data. The quality of the raw sequenced data was initially examined using FastQC V0.11.5. The 5′ adapter GTTCAGAGTTCTACAGTCCGACGATC, the 3′ adapter AACTGTAGGCACCATCAAT, and sequences below 15 nucleotides in length were removed in the pre-processing step. The Illumina TruSeq stop oligo sequences were also removed. sRNAnalyzer uses Cutadapt [14] to trim the adapter sequences and uses the software Prinseq [15] to remove low quality and short reads (nt < 15). The quality of the pre-processed data was examined using the multiqc software [16]. The alignment module in sRNAnalyzer uses Bowtie [17] for alignment and provides choices for various miRNA and small RNA databases for mapping. For this study, miRBase (v21) and miRNA precursor annotations in MirGeneDB [18] with a single read assignment option in which each read is counted only once to its first best alignment were used. The alignment module uses a local probabilistic model to find the best possible assignment of those reads that have multiple matches. The counts for individual mature or precursor miRNA were obtained using the summarisation module.

### Statistical methodology for differential expression analysis

Sequencing count data were normalised and miRNAs with more than 30% missing observations were excluded. Additionally, very lowly expressed miRNAs (< 5 for more than 10% of the samples) and those with very low average counts (< 5) were excluded from the analysis. All the differential expression analyses including the normalisation of read counts and comparison of two groups were done using the Bioconductor package edgeR [19]. Differential miRNA expression was examined between the amyloid-positive AD and amyloid-negative control groups using the Fisher’s exact test in edgeR. A *p* value of < 0.05 was considered nominally significant and because of the high number of tests performed, all *p* values were corrected for multiple testing. A false discovery rate (FDR) of < 0.1 was considered significant for this study. The FDR *p* values were obtained using the Benjamini and Hochberg (1995) procedure as implemented in edgeR [20]. The receiver operating characteristic curve (ROC) and the area under the ROC (AUC) based on the logistic regression analysis of case-control status with the set of predictors were obtained using the R package pROC [21].

### Replication of results in an independent cohort

Replication of significantly differentiated miRNAs between AD and cognitively normal controls in the discovery AIBL cohort was performed in an independent sample with previously published miRNA data by Leidinger and colleagues [22]. The AD group fulfilled the NINCDS-ADRDA criteria for probable AD, but this study cohort was not biomarker-defined with brain amyloid imaging. The raw small-RNA sequencing data from this cohort were downloaded as FASTQ-formatted files from the DDBJ Sequence Read Archive (DRA) (trace.ddbj.nig.ac.jp/DRASearch) under the accession number SRP022043. The data were processed using the same pipeline as this current study and differential miRNA expression analyses between AD and control groups performed as outlined above.

### MiRNA target and biological function analyses

Candidate replicated miRNAs were assessed for putative target genes and pathways. Using Ingenuity® Pathway Analysis (IPA) (www.ingenuity.com), putative target genes were identified using the ‘microRNA Target Filter’ tool. Only target genes which were predicted in silico with high confidence and/or were experimentally validated were included. Gene-Enrichment/Functional Annotation analysis for the lists of mRNA targets was conducted using DAVID (the database for annotation, visualisation and integrated discovery; https://david.ncifcrf.gov/home.jsp) bioinformatics resource 6.8 [23]. DAVID functional annotation clustering was performed for each target mRNA gene list derived from each selected candidate miRNA (using IPA miRNA target filter tool described above). Relevant pathways using DAVID pathway viewer was accessed where applicable when there was an overrepresentation of genes in the selected lists in a relevant KEGG (Kyoto Encyclopedia of Genes and Genomes) pathway [24].

## Results

### AIBL cohort selection and demographics

From the AIBL cohort, 80 participants (45 amyloid positive AD and 35 controls) had PAXgene tubes of whole blood available for RNA extraction. Following RNA extraction, 9 samples were excluded due to low RNA integrity number (RIN) of less than 6.5. Subsequently, 71 samples (40 AD and 31 controls) underwent miRNA profiling. The sample demographics are shown in Table 1*.* The mean age and standard deviation (SD) for the cohort (*n* = 71) was 73.0 ± 6.3. The mean Mini-Mental State Examination (MMSE) [25] score for the AD group was 21.1 (SD 4.6), and for the control group 29.1 (SD 1.0). There was an overrepresentation of individuals with one or more *APOE* Ɛ4 alleles in the AD (80%) group, with the control group having only 32% positive for the presence of an *APOE* Ɛ4 allele.

**Table 1:** Cohort demographics

Sample group	*N*	Age (mean ± SD)	Sex (m/f)	MMSE (mean ± SD)	Amyloid PET status	*APOE* Ɛ4 carrier, *N* (%)
Whole cohort	71	73.0 ± 6.3	33/38	24.6 ± 5.3	n/a	n/a
AD	40	74.9 ± 6.0	19/21	21.1 ± 4.6	Positive	32 (80%)
Controls	31	71.0 + 5.9	14/17	29.1 ± 1.0	Negative	10 (32%)

### Differential miRNA analysis: AD versus controls

Following normalisation and quality control checks, 816 unique miRNAs were examined between individuals with amyloid-positive AD and amyloid-negative controls. There were 71 miRNAs differentially expressed between the two groups (*p* < 0.05) and four of these remained significant after controlling for multiple testing (FDR < 0.1). The 71 differentially expressed miRNAs are shown in Table 2*.* Of these, 43 miRNAs were upregulated and 28 were downregulated in AD compared to controls. The top FDR significant miRNA (hsa-miR-218-1-5p) had a high fold change of 7.15. The magnitude of fold change for the remaining miRNAs ranged from 0.34 to 2.90. In addition, there were no significant changes to the results shown in Table 2 after adjusting for the covariates of age, sex, education, and MMSE (Supplementary Table 1).

**Table 2:** Dysregulated miRNAs between amyloid-positive AD and amyloid-negative cognitively normal controls

MiRNA	Log_2_ FC (AD vs controls)	Fold change^a^	*p* value (unadj)	*p* value (FDR-corrected)
hsa-miR-218-1-5p	2.84	7.15	5.32E-08	4.34E-05
hsa-miR-4482-3p	− 1.54	0.34	8.79E-05	0.035865
hsa-miR-16-2-3p	0.78	1.72	0.000409	0.095074
hsa-miR-4669-3p	1.30	2.45	0.000466	0.095074
hsa-let-7b-5p	0.75	1.69	0.000771	0.125787
hsa-miR-320a-3p	0.56	1.47	0.001074	0.132259
hsa-miR-574-5p	0.78	1.71	0.001135	0.132259
hsa-miR-5010-5p	0.72	1.65	0.001754	0.178899
hsa-miR-181a-1-5p	0.46	1.38	0.004002	0.277468
hsa-miR-1306-3p	0.68	1.60	0.004171	0.277468
hsa-miR-320c-1-3p	0.53	1.45	0.004248	0.277468
hsa-miR-3682-3p	0.68	1.60	0.004318	0.277468
hsa-miR-7113-5p	0.67	1.59	0.004566	0.277468
hsa-miR-30a-5p	0.67	1.59	0.00476	0.277468
hsa-miR-6793-3p	0.50	1.41	0.005823	0.316788
hsa-miR-3135a-5p	0.60	1.51	0.006553	0.328554
hsa-miR-548ae-2-5p	1.10	2.14	0.007103	0.328554
hsa-mir-3138	− 1.14	0.45	0.007248	0.328554
hsa-miR-6884-5p	0.58	1.49	0.008981	0.385731
hsa-miR-25-5p	0.44	1.36	0.010531	0.404056
hsa-miR-320b-1-3p	0.43	1.35	0.010797	0.404056
hsa-miR-4772-3p	− 0.60	0.66	0.011461	0.404056
hsa-miR-3064-5p	0.56	1.47	0.011532	0.404056
hsa-mir-3607	− 0.53	0.69	0.012026	0.404056
hsa-miR-421-3p	− 0.25	0.84	0.013088	0.404056
hsa-mir-3922	0.50	1.42	0.013536	0.404056
hsa-miR-3605-5p	0.46	1.37	0.014781	0.404056
hsa-miR-4649-3p	− 0.53	0.69	0.01502	0.404056
hsa-miR-30a-3p	0.67	1.59	0.015235	0.404056
hsa-miR-542-3p	0.54	1.46	0.015267	0.404056
hsa-miR-4732-5p	0.42	1.34	0.01535	0.404056
hsa-miR-337-3p	− 0.51	0.70	0.016418	0.412266
hsa-miR-320d-1-3p	0.47	1.38	0.017108	0.412266
hsa-mir-1273 g	0.36	1.29	0.017178	0.412266
hsa-miR-3913-1-3p	− 0.34	0.79	0.018048	0.420787
hsa-miR-3691-3p	− 0.35	0.78	0.020256	0.459135
hsa-miR-5701-1-5p	− 0.57	0.67	0.023849	0.475508
hsa-miR-589-3p	− 0.26	0.84	0.023928	0.475508
hsa-mir-202	− 0.62	0.65	0.02398	0.475508
hsa-miR-23b-5p	0.33	1.26	0.02412	0.475508
hsa-miR-3138-3p	0.46	1.38	0.024643	0.475508
hsa-miR-641-5p	− 0.32	0.80	0.025083	0.475508
hsa-miR-15b-5p	− 0.25	0.84	0.025333	0.475508
hsa-mir-1248	− 0.62	0.65	0.026133	0.475508
hsa-miR-664b-5p	0.43	1.34	0.026223	0.475508
hsa-miR-579-5p	0.33	1.26	0.027033	0.479535
hsa-miR-2277-3p	0.37	1.29	0.027747	0.481733
hsa-miR-1287-5p	0.47	1.38	0.031054	0.525366
hsa-miR-627-3p	− 0.29	0.82	0.032362	0.525366
hsa-miR-423-5p	0.30	1.23	0.032829	0.525366
hsa-miR-92b-5p	0.41	1.33	0.032835	0.525366
hsa-miR-548n-3p	− 0.40	0.76	0.035717	0.541575
hsa-miR-3607-3p	− 0.51	0.70	0.035911	0.541575
hsa-miR-146b-5p	− 0.29	0.82	0.036279	0.541575
hsa-miR-3667-5p	0.59	1.50	0.03678	0.541575
hsa-miR-185-3p	0.31	1.24	0.03858	0.541575
hsa-miR-10a-5p	− 0.41	0.75	0.038723	0.541575
hsa-miR-628-5p	− 0.30	0.81	0.039484	0.541575
hsa-miR-190a-5p	0.52	1.44	0.04009	0.541575
hsa-miR-1284-5p	− 0.24	0.84	0.04149	0.541575
hsa-let-7a-3	0.50	1.41	0.042166	0.541575
hsa-miR-4742-3p	− 0.32	0.80	0.04264	0.541575
hsa-miR-196a-1-5p	0.80	1.74	0.04283	0.541575
hsa-miR-6513-3p	− 0.25	0.84	0.043181	0.541575
hsa-miR-1294-5p	0.39	1.31	0.043499	0.541575
hsa-miR-3130-1-5p	− 0.27	0.83	0.043804	0.541575
hsa-miR-643-3p	− 0.38	0.77	0.04469	0.544284
hsa-miR-6729-3p	0.40	1.32	0.045509	0.546106
hsa-mir-1229	0.48	1.40	0.046396	0.548684
hsa-miR-654-3p	− 0.46	0.73	0.048297	0.563006
hsa-miR-128-1-3p	− 0.23	0.85	0.049922	0.56594

*Log*_*2*_*FC* log_2_ fold change, directionality denoted by + (upregulated) or – (downregulated) value when comparing AD to controls

^a^Magnitude of fold change between AD compared to controls

### Replication of dysregulated miRNAs in an independent cohort

Using the same bioinformatics pipeline as the original analysis of differential blood miRNA expression in amyloid-positive AD and amyloid-negative controls, an independent blood miRNA-seq dataset derived from 48 AD and 22 cognitively normal control individuals was used to replicate the identified significant miRNAs shown in Table 2 [22]. This cohort comprised an AD group of 23 males and 25 females with average age 70.3 ± 7.9 years and MMSE score of 18.7 ± 3.5, as well as a normal control group of 11 males and 11 females with average age 67.1 ± 7.5 years and MMSE score 29.3 ± 1.2. In this cohort, 567 mature miRNAs were examined following data cleaning and normalisation using our bioinformatics pipeline. Of the 816 miRNAs examined in our AIBL cohort, 478 of these overlapped with miRNAs examined in the replication cohort.

**Fig. 1:**
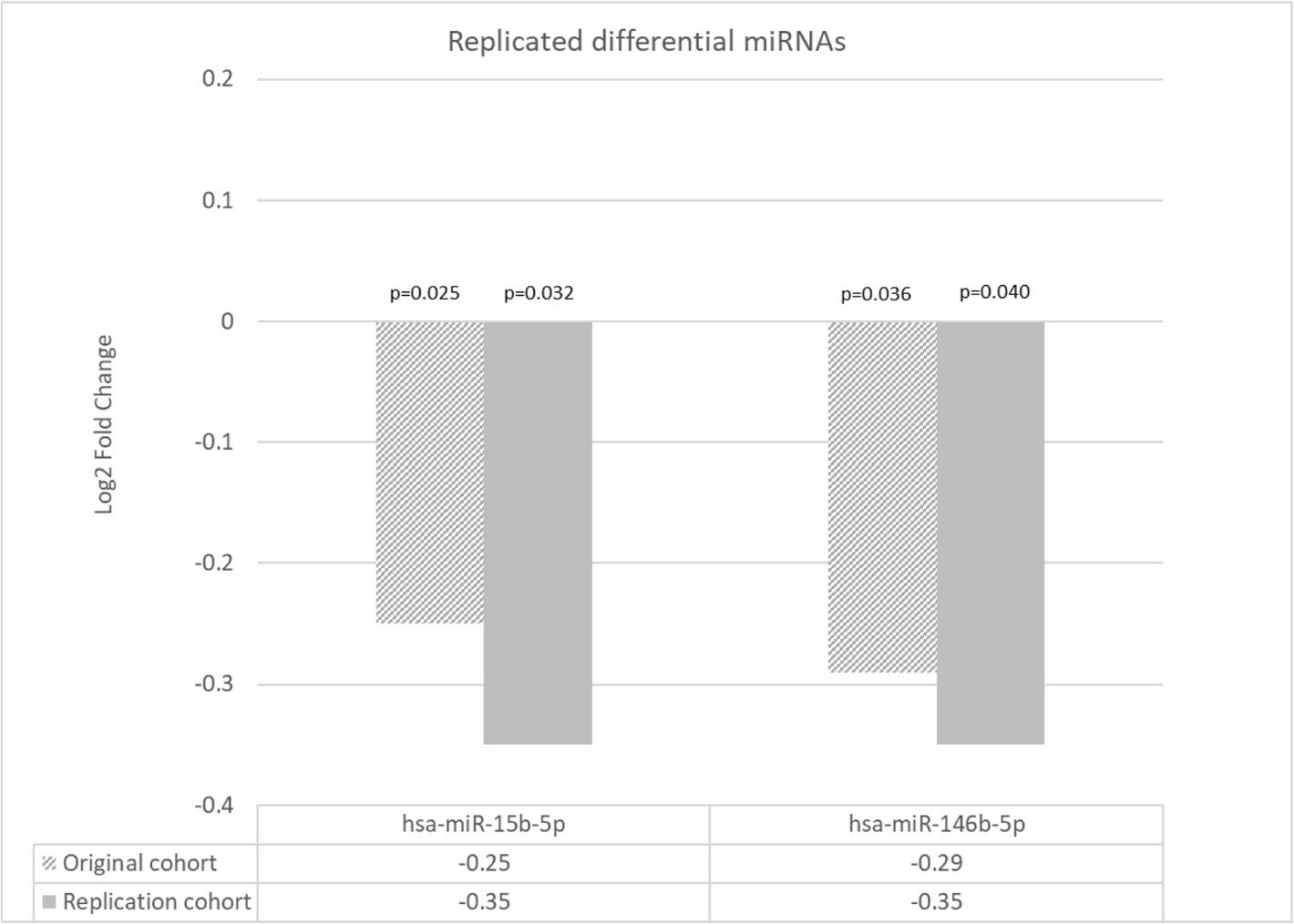
Replicated significant miRNAs identified in the current study (AIBL cohort) in an independent cohort [22]. logFC = log_2_ fold change, directionality denoted by + (upregulated) or – (downregulated) value when comparing AD to controls

In the replication cohort, there were 163 differentially expressed miRNAs between AD and controls with FDR-corrected *p* value < 0.1 and 191 differentially expressed miRNAs with unadjusted *p* value of 0.05. The results are presented in Supplementary Table 2*.* Of the original 71 differentially expressed miRNAs between AD and controls in the AIBL cohort, there were 34 miRNAs overlapping, of which only two miRNAs were significantly differentially expressed (*p* < 0.05) and in the same direction of dysregulation (Fig. 1). MiR-15b-5p was downregulated in AD compared to controls in both the original cohort and in the replication cohort with fold change 0.84 and 0.78 (log2 fold change − 0.25 and − 0.35), respectively. Similarly, miR-146b-5p was downregulated in AD compared to controls with fold changes of 0.82 and 0.78 (log2 fold change − 0.29 and − 0.35), respectively. To identify any relationships with demographic measures, correlation analyses were performed in the AIBL cohort for miR-15b-5p and miR-146b-5p with age, sex, years of education, and MMSE. There were no significant correlations identified between the two miRNAs and the above demographic variables (*p* > 0.05).

**Fig. 2:**
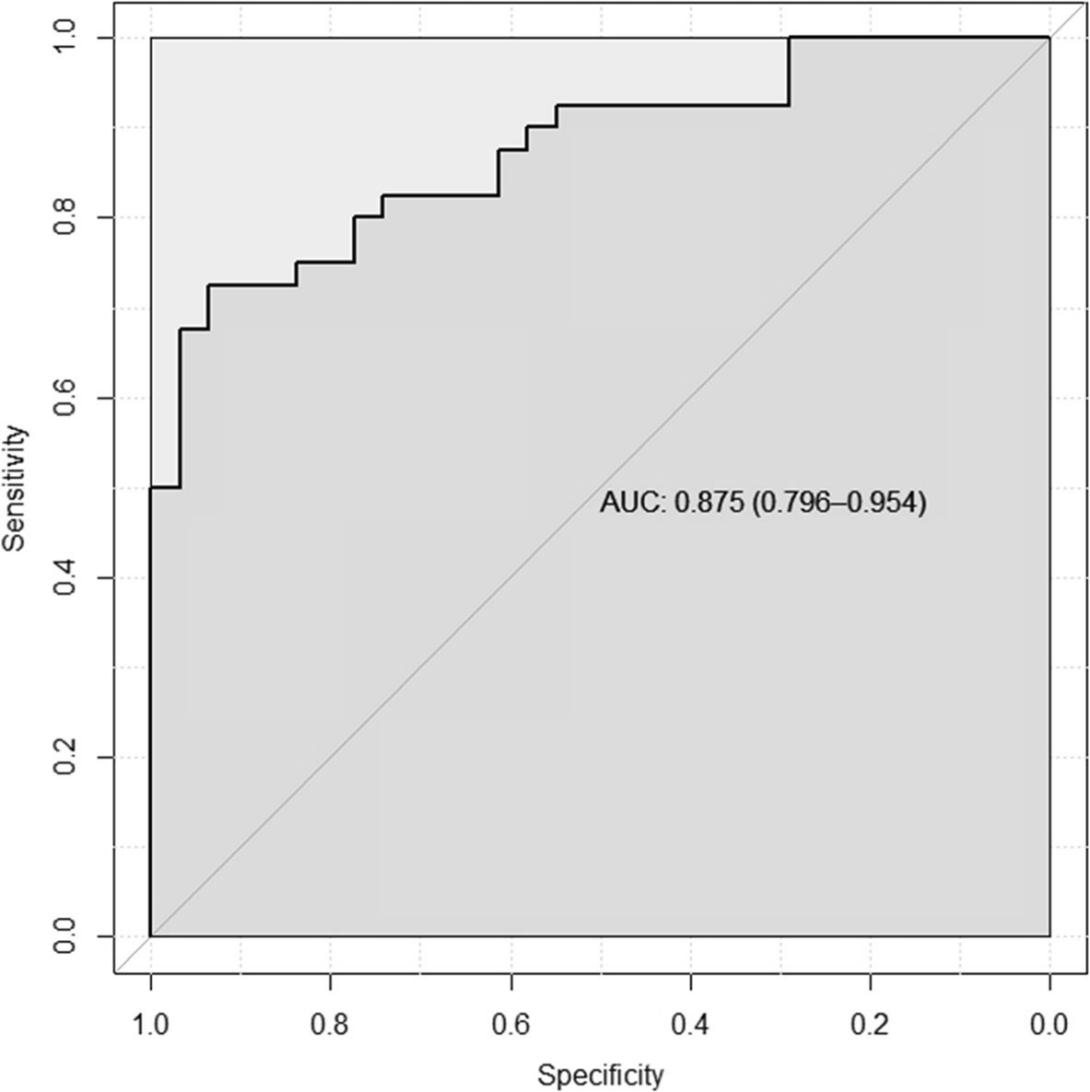
ROC and AUC based on the predictors’ age, sex, education, APOE4 carrier status, and the two microRNAs: miR-15b-5p and miR146b-5p

A ROC analysis was performed for the two replicated miRNAs, miR-15b-5p and miR-146b-5p. ROC fit was based on a model using these two miRNAs and the demographic predictors of age, sex, education, and APOE4 carrier status. The area under the curve (AUC) was 0.875 with a 95% bootstrap confidence interval of 0.796–0.954 (Fig. 2).

### MiRNA target analysis

Target mRNA information for miR-15b-5p and miR-146b-5p were sought bioinformatically (IPA). Results were filtered to include only experimentally validated or highly predicted targets. MiR-146b-5p had 158 mRNA targets fulfilling these criteria, and miR-15b-5p had 526 mRNA targets. To assess the biological function of these target mRNAs, gene enrichment functional annotation analysis using DAVID 6.8 was performed. Messenger RNA gene lists of miR-15b-5p and miR-146b-5p were uploaded for functional analysis. The results of the functional annotation analysis are summarised in Table 3, with the top annotation clusters with FDR significance listed. Messenger RNA targets of miR-146b-5p were centred around the innate immune system and cytokine pathways. MiR-15b-5p appears to target mRNAs involved in cell cycle and apoptosis. Pathways analysis showed the mRNAs targeted by miR-146b-5p to be involved in the toll-like receptor signalling pathway, with 14 mRNAs (9.2%) of the target list involved. This is consistent with the results of annotation clustering as this pathway is critical to the innate response system. Cancer pathways feature predominantly for miR-15b-5p, consistent with its annotation clustering of apoptosis and regulation of cell cycle.

**Table 3:** Functional annotation analysis of predicted gene targets of identified candidate miRNAs

Candidate miRNA	Number of annotation clusters with enrichment score > 1.3 for predicted gene targets	Annotation cluster	Gene count*	Enrichment score	*p* value	FDR
miR-146b-5p	34	Innate immune response	24	13.8	6.1E−13	3.0E−10
		Cellular response to cytokine stimulus	33	11.72	1.9E−15	3.21E−12
		Defence response to bacterium	25	9.43	2.4E−12	4.03E−09
		Interleukin-1 receptor binding	7	6.35	3.66E−10	4.83E−07
		Regulation of interleukin-6 production	13	4	7.4E−11	1.26E−07
		Regulation of interleukin-12 production	7	3.87	3.89E−06	0.006623
		Lipopolysaccharide-mediated signalling pathway	8	3.82	1.50E−07	2.55E−04
		Regulation of cytokine secretion	10	3.68	4.44E−06	0.007556
miR-15b-5p	31	Apoptotic process	91	6.08	2.2E−9	4.04E−06
		Regulation of protein metabolic process	119	5.71	9.9E−10	1.80E−06
		Regulation of cell cycle	36	2.55	3.2E−6	0.005875

Functional annotation clustering analysis of target mRNA gene lists of candidate miRNAs using DAVID. Top annotation clusters are listed

*Number of genes from the target mRNA gene list submitted for each miRNA (158 genes for miR-146b-5p and 526 genes for miR-15b-5p) that are involved in the annotation cluster

## Discussion

Prior studies seeking to identify miRNA blood biomarkers for AD have had inconsistent results, with most candidates lacking replication across independent cohorts. One factor contributing to the variation in results across studies is the different phenotypic definitions used to classify participant groups. This includes potential inclusion of controls that may have pre-clinical AD or the use of AD participants that may not have AD pathology. To minimise this issue in our study, participants were selected with the aid of brain amyloid burden data. This study, with the use of a cohort of amyloid imaging-defined phenotypes in combination with robust longitudinal data, is one of the most strictly phenotyped AD biomarker studies to date.

### Differentially expressed miRNAs between AD and controls

Using a non-hypothesis driven, 71 differentially expressed miRNAs were found between AD and controls. In a replication cohort, utilising a publicly available dataset and applying our bioinformatics pipeline, two of the original 71 differentially expressed miRNAs were replicated (miR-15b-5p and miR-146b-5p). We propose these two miRNAs as candidate biomarkers for AD for further investigation. Indeed, there is already evidence from existing literature of their ability to differentiate AD from cognitively normal controls. MiR-15b-5p has been previously shown to be downregulated in the blood of individuals with AD compared to controls, with a reported sensitivity of 0.85, specificity of 0.88, and area under the curve (AUC) of 0.96 in differentiating those with AD from controls [5]. In another study examining differential blood miRNA expression among discordant twins (*n* = 22 twin pairs) for dementia (including AD, vascular, or unspecified), miR-146b-5p was downregulated in twins who were diagnosed with dementia [26]. Although this study was not specific for AD, the use of the discordant MZ twin model is a powerful tool for epigenetic studies as it controls for potential confounders encountered in case-control studies, such as differences in genetic factors, age, gender, maternal effects, and most in utero and early environmental influences.

### Messenger RNA targets and biological function analysis

To further our understanding of the biological function of the two candidate miRNA biomarkers and their potential role in AD pathology, in silico analysis of the target mRNAs of miR-146b-5p and miR-15b-5p was performed. Annotation clustering for miRNA-146b-5p gene targets was found to be centred around the innate immune system and cytokine production. This was further supported by the overrepresentation of those genes in the KEGG pathway for the toll-like receptor signalling pathway, a crucial pathway involved in the innate immune response. Interestingly, in recent years, neuroinflammation has been recognised as an important component of AD pathology. Experimental, genetic and epidemiological evidence now indicate a crucial role for activation of the innate immune system as a disease-promoting factor [27]. Annotation clustering for miRNA-15b-5p gene targets showed they are involved in apoptosis and regulation of the cell cycle. Pathway analysis of these mRNAs showed involvement in a number of cancer pathways. The relationship between cancer and AD has been of great interest to the research community. An inverse association between AD and cancer has been previously noted, with AD individuals developing cancer at a slower rate than the general population, and those with a history of cancer developing AD at a slower rate [28]. The pathomechanism is not clear, but it is postulated that in cancer, cell regulation mechanisms are disrupted with augmentation of cell survival or proliferation, whereas conversely, AD is associated with increased neuronal death, driven by beta amyloid (Aβ) and tau deposition [29]. As discussed in a review by Holohan et al., a number of dysregulated miRNAs have been identified in both AD and cancer, suggesting that miRNAs play multiple regulatory roles in pathways active across both cancer and AD [30]. Interestingly, miR-15b-5p has been reported to promote gastric cancer metastasis and to be upregulated in gastric cancer cell lines, tissues, and plasma samples [31]. In this AD cohort, miR-15b-5p was downregulated in AD compared to controls. Despite these postulations, future studies into the biological role of miRNAs will need further targeted experiments to confirm the validity and the biological significance of the identified in silico and experimental miRNA-mRNA interactions.

### Limitations of study and future direction

This study has several limitations. Firstly, despite using the same bioinformatics pipeline, only two of the original 71 differentially expressed miRNAs were similarly dysregulated in the replication cohort. Reasons for this are largely attributable to the differences in study cohorts. The case-control phenotypes in the current study were defined by amyloid imaging, whereas the replication cohort did not have amyloid biomarker support. There were further differences in disease severity and demographics of the two cohorts, including age (older in the present study) and MMSE (mean MMSE in replication AD sample being 18.9 (SD ± 3.4) compared to 21.1 (SD ± 4.6) in the present study).

Secondly, only one of the many bioinformatics methods available was used to analyse results (Edge-R). Indeed, it is well recognised that different statistical methods will result in different significant miRNAs. When the full results obtained in the replication cohort using our bioinformatics pipeline (data not shown) were compared to the results published by the original investigators using their methodology, only 45 significant miRNAs overlapped. The discrepancy has also been demonstrated by Satoh and colleagues, who took the same publicly available dataset from Leidinger et al. and processed the FASTQ files with a different bioinformatics pipeline [32]. Their pipeline yielded only 27 differentially expressed miRNAs between AD and controls, and of which only two were reported to be significant by the original investigators. There is an urgent need for consistency of data analysis across studies; however, this is problematic as there are no best practice methods and consensus for the most appropriate method. Moreover, a new methodology is continually being published, thus confounding the comparison of results across different studies.

Thirdly, our results were not experimentally validated using a different technology such as q-PCR, although replication in independent cohorts is a more robust approach to strengthening candidate miRNA biomarker findings, which was performed here using data from a previously published study. Finally, the expression of miRNA blood may not reflect the changes occurring in the target organ—the brain. MiRNA-146b-5p and miR-15b-5p are not brain- or AD-specific miRNAs. Therefore, these differentially expressed miRNAs need further experimental validation to elucidate their role in pathobiology and effects at the target organ.

## Conclusion

Two miRNAs identified in this study, miR-146b-5p and miR15b-5p, showed a consistent relative change in expression levels between AD and controls. Their biological function may also be related to AD pathogenesis. Further replication studies are needed to establish their role as biomarkers for AD, including determining absolute value cut-offs and sensitivity/specificity analyses. To minimise differences in methodologies confounding results when comparing across studies, large consortia cohorts using a consensus approach are best positioned to drive further translational studies. Experimental validation of mRNA targets and further elucidation of the role these miRNAs play in biology may harbour insights into AD pathogenesis and potential therapeutic targets.

## Supplementary information

**Additional file 1 : Supplementary Table 1.** Dysregulated miRNAs between amyloid positive AD and amyloid negative cognitively normal controls adjusted for age, sex, years of education, and MMSE score. **Supplementary Table 2.** Results of nominally significant (*p*<0.05) differential miRNA expression between AD and controls in the validation set (Leidinger cohort).

## Data Availability

The datasets used and/or analysed during the current study are available from the corresponding author on reasonable request.

## Abbreviations

3′ UTR: 3 prime untranslated region
AD: Alzheimer’s disease
ADRDA: Alzheimer’s Disease and Related Disorders Association
AIBL: Australian Imaging Biomarkers Lifestyle Study of Ageing
APOE: Apolipoprotein E
Aβ: Beta-amyloid
CSF: Cerebrospinal fluid
DAVID: Database for Annotation, Visualisation and Integrated Discovery
DNA: Deoxyribonucleic acid
FC: Fold change
FDR: False discovery rate
hsa: *Homo sapiens*
IPA: Ingenuity Pathway Analysis
KEGG: Kyoto Encyclopedia of Genes and Genomes
MCI: Mild cognitive impairment
miRNA: MicroRNA
MMSE: Mini-mental State Examination
MRI: Magnetic resonance imaging
mRNA: Messenger RNA
N/A: Not applicable or not available
NIA-AA: National Institute on Aging and Alzheimer’s Association
NINCDS: The National Institute of Neurological and Communicative Disorders and Stroke
PET: Positron emission tomography
qPCR: Quantitative real-time PCR
RIN: RNA integrity number
RNA: Ribonucleic acid
SD: Standard deviation
SUVR: Standardised uptake value ratio
