# Supplementary material for: Differential blood miRNA expression in brain amyloid imaging-defined Alzheimer’s disease and controls
Source: Alzheimers Res Ther. 2020 May 15;12:59. doi: 10.1186/s13195-020-00627-0 (PMC7229622; doi:10.1186/s13195-020-00627-0)
Supplement: Supplementary file 1 — Additional file 1 : Supplementary Table 1. Dysregulated miRNAs between amyloid positive AD and amyloid negative cognitively normal controls adjusted for age, sex, years of education, and MMSE score. Supplementary Table 2. Results of nominally significant (p<0.05) differential miRNA expression between AD and controls in the validation set (Leidinger cohort). [file 13195_2020_627_MOESM1_ESM.docx]

Supplementary Table 1: *Dysregulated miRNAs between amyloid positive AD and amyloid negative cognitively normal controls adjusted for age, sex, years of education, and MMSE score.*

| miRNA | Log_2_ FC (AD vs controls) | Fold change^a^ | p-value (unadj) | p-value (FDR- corrected) |
| --- | --- | --- | --- | --- |
| hsa-miR-218-1-5p | 2.839326 | 7.156856 | 9.30E-10 | 7.59E-07 |
| hsa-miR-4482-3p | -1.53589 | 0.344868 | 8.86E-05 | 0.036159 |
| hsa-miR-16-2-3p | 0.784488 | 1.722481 | 0.000340372 | 0.092581 |
| hsa-miR-4669-3p | 1.295407 | 2.454463 | 0.00046656 | 0.095178 |
| hsa-let-7b-5p | 0.753147 | 1.685465 | 0.0007779 | 0.126953 |
| hsa-miR-320a-3p | 0.556594 | 1.470792 | 0.001229478 | 0.161015 |
| hsa-miR-574-5p | 0.778197 | 1.714986 | 0.001381256 | 0.161015 |
| hsa-miR-5010-5p | 0.721519 | 1.648917 | 0.001860153 | 0.189736 |
| hsa-miR-548ae-2-5p | 1.096097 | 2.137756 | 0.003152827 | 0.256384 |
| hsa-miR-1306-3p | 0.675587 | 1.597246 | 0.003179693 | 0.256384 |
| hsa-miR-3682-3p | 0.675468 | 1.597115 | 0.004234291 | 0.256384 |
| hsa-miR-181a-1-5p | 0.464135 | 1.37949 | 0.004268437 | 0.256384 |
| hsa-miR-7113-5p | 0.667542 | 1.588365 | 0.004340979 | 0.256384 |
| hsa-mir-3138 | -1.13828 | 0.4543 | 0.004465268 | 0.256384 |
| hsa-miR-320c-1-3p | 0.532564 | 1.446498 | 0.004861659 | 0.256384 |
| hsa-miR-3135a-5p | 0.597721 | 1.513324 | 0.005337009 | 0.256384 |
| hsa-miR-30a-5p | 0.668026 | 1.588897 | 0.005341335 | 0.256384 |
| hsa-miR-6793-3p | 0.50016 | 1.414371 | 0.005835167 | 0.264528 |
| hsa-miR-6884-5p | 0.578426 | 1.493219 | 0.008520473 | 0.365932 |
| hsa-miR-4772-3p | -0.59622 | 0.661484 | 0.010505115 | 0.400929 |
| hsa-miR-25-5p | 0.441876 | 1.35837 | 0.011125993 | 0.400929 |
| hsa-mir-3922 | 0.502449 | 1.416616 | 0.011808446 | 0.400929 |
| hsa-miR-320b-1-3p | 0.428695 | 1.346015 | 0.011953628 | 0.400929 |
| hsa-miR-3064-5p | 0.560351 | 1.474628 | 0.012098433 | 0.400929 |
| hsa-mir-3607 | -0.53058 | 0.692277 | 0.013065544 | 0.400929 |
| hsa-miR-542-3p | 0.542276 | 1.456268 | 0.013265243 | 0.400929 |
| hsa-miR-337-3p | -0.51351 | 0.700516 | 0.013611098 | 0.400929 |
| hsa-miR-421-3p | -0.24826 | 0.841909 | 0.013849029 | 0.400929 |
| hsa-miR-3605-5p | 0.455437 | 1.371198 | 0.01424872 | 0.400929 |
| hsa-miR-4649-3p | -0.5296 | 0.692746 | 0.015289626 | 0.415878 |
| hsa-miR-30a-3p | 0.667428 | 1.588239 | 0.015864679 | 0.416863 |
| hsa-miR-4732-5p | 0.420492 | 1.338384 | 0.016347586 | 0.416863 |
| hsa-miR-3913-1-3p | -0.33767 | 0.79132 | 0.017279989 | 0.427287 |
| hsa-mir-1273g | 0.363701 | 1.286723 | 0.018381832 | 0.441164 |
| hsa-miR-320d-1-3p | 0.46603 | 1.381304 | 0.019092676 | 0.445132 |
| hsa-miR-3691-3p | -0.35172 | 0.783648 | 0.02184764 | 0.484199 |
| hsa-miR-579-5p | 0.32798 | 1.255255 | 0.022755636 | 0.484199 |
| hsa-mir-202 | -0.61623 | 0.652375 | 0.025736332 | 0.484199 |
| hsa-miR-589-3p | -0.25889 | 0.835731 | 0.025892502 | 0.484199 |
| hsa-miR-5701-1-5p | -0.5691 | 0.674036 | 0.025921222 | 0.484199 |
| hsa-miR-3138-3p | 0.462661 | 1.378081 | 0.025950555 | 0.484199 |
| hsa-miR-641-5p | -0.31818 | 0.80208 | 0.026219645 | 0.484199 |
| hsa-mir-1248 | -0.62323 | 0.649214 | 0.026392335 | 0.484199 |
| hsa-miR-23b-5p | 0.335429 | 1.261753 | 0.02653249 | 0.484199 |
| hsa-miR-15b-5p | -0.24961 | 0.841124 | 0.026795159 | 0.484199 |
| miRNA | Log_2_ FC (AD vs controls) | Fold change^a^ | p-value (unadj) | p-value (FDR- corrected) |
| hsa-miR-664b-5p | 0.427929 | 1.345301 | 0.027295525 | 0.484199 |
| hsa-miR-2277-3p | 0.372908 | 1.294961 | 0.02976354 | 0.516746 |
| hsa-miR-627-3p | -0.28849 | 0.81876 | 0.031943226 | 0.535466 |
| hsa-miR-3667-5p | 0.587983 | 1.503144 | 0.033852776 | 0.535466 |
| hsa-miR-1287-5p | 0.465381 | 1.380681 | 0.033922224 | 0.535466 |
| hsa-miR-548n-3p | -0.40307 | 0.756245 | 0.034458939 | 0.535466 |
| hsa-miR-92b-5p | 0.411232 | 1.329821 | 0.034826175 | 0.535466 |
| hsa-miR-423-5p | 0.297479 | 1.228995 | 0.036493627 | 0.535466 |
| hsa-mir-1229 | 0.484603 | 1.399201 | 0.036907213 | 0.535466 |
| hsa-miR-10a-5p | -0.40977 | 0.752745 | 0.037445324 | 0.535466 |
| hsa-miR-190a-5p | 0.524247 | 1.438183 | 0.037449103 | 0.535466 |
| hsa-miR-185-3p | 0.313811 | 1.242987 | 0.039287007 | 0.535466 |
| hsa-miR-146b-5p | -0.28675 | 0.819748 | 0.040153784 | 0.535466 |
| hsa-let-7a-3 | 0.497133 | 1.411406 | 0.041181942 | 0.535466 |
| hsa-miR-3607-3p | -0.5136 | 0.700472 | 0.041287959 | 0.535466 |
| hsa-miR-628-5p | -0.29921 | 0.812699 | 0.041296027 | 0.535466 |
| hsa-miR-6729-3p | 0.398553 | 1.318185 | 0.041351676 | 0.535466 |
| hsa-miR-6513-3p | -0.25031 | 0.840718 | 0.04225384 | 0.535466 |
| hsa-miR-196a-1-5p | 0.795989 | 1.736267 | 0.042255155 | 0.535466 |
| hsa-miR-1284-5p | -0.24418 | 0.844293 | 0.04346177 | 0.535466 |
| hsa-let-7i-3p | 0.191766 | 1.142161 | 0.044010831 | 0.535466 |
| hsa-miR-128-1-3p | -0.22949 | 0.852935 | 0.044483937 | 0.535466 |
| hsa-miR-643-3p | -0.37631 | 0.770406 | 0.044622183 | 0.535466 |
| hsa-miR-654-3p | -0.45729 | 0.728352 | 0.046078352 | 0.544927 |
| hsa-miR-4742-3p | -0.32415 | 0.798767 | 0.047321773 | 0.546545 |
| hsa-miR-1294-5p | 0.389228 | 1.309692 | 0.047646913 | 0.546545 |
| hsa-miR-3130-1-5p | -0.26814 | 0.830392 | 0.048224528 | 0.546545 |

Log_2_FC = log_2_ fold change, directionality denoted by + (upregulated) or – (downregulated) value when comparing AD to controls. ^a^ Magnitude of fold change between AD compared to controls

*Supplementary Table 2: Results of nominally significant (p<0.05) differential miRNA expression between AD and controls in the validation set (Leidinger cohort)*

| miRNA | logFC | PValue | FDR |
| --- | --- | --- | --- |
| hsa-miR-6131-3p | -2.20 | 2.27E-12 | 1.29E-09 |
| hsa-miR-3200-3p | -1.74 | 9.92E-12 | 2.81E-09 |
| hsa-miR-3135b-5p | -1.63 | 5.44E-10 | 1.03E-07 |
| hsa-let-7f-1-5p | -1.12 | 9.87E-09 | 1.40E-06 |
| hsa-miR-15a-5p | -1.45 | 2.21E-08 | 2.50E-06 |
| hsa-miR-148a-3p | -1.59 | 6.83E-08 | 6.46E-06 |
| hsa-miR-199a-1-3p | -1.42 | 1.17E-07 | 9.44E-06 |
| hsa-miR-144-5p | -1.72 | 1.33E-07 | 9.45E-06 |
| hsa-miR-4781-3p | 0.96 | 1.59E-07 | 1.00E-05 |
| hsa-miR-17-3p | -1.44 | 1.88E-07 | 1.06E-05 |
| hsa-miR-548aj-2-5p | -1.68 | 3.13E-07 | 1.61E-05 |
| hsa-mir-194-1 | 4.39 | 3.48E-07 | 1.64E-05 |
| hsa-miR-98-5p | -1.07 | 3.86E-07 | 1.69E-05 |
| hsa-miR-1468-5p | 1.25 | 7.99E-07 | 3.24E-05 |
| hsa-miR-660-5p | -1.47 | 9.66E-07 | 3.53E-05 |
| hsa-let-7g-5p | -1.10 | 1.03E-06 | 3.53E-05 |
| hsa-miR-26b-3p | 0.97 | 1.06E-06 | 3.53E-05 |
| hsa-miR-1294-5p | -1.12 | 2.43E-06 | 7.66E-05 |
| hsa-miR-151a-3p | 0.75 | 7.67E-06 | 0.000229 |
| hsa-miR-5001-3p | 0.87 | 8.43E-06 | 0.000232 |
| hsa-miR-29c-3p | -1.29 | 8.60E-06 | 0.000232 |
| hsa-miR-6803-3p | -0.90 | 1.32E-05 | 0.00034 |
| hsa-miR-151b-3p | 1.24 | 3.25E-05 | 0.000801 |
| hsa-miR-6790-3p | -1.12 | 3.76E-05 | 0.000888 |
| hsa-miR-148b-5p | 0.85 | 3.98E-05 | 0.000904 |
| hsa-miR-361-5p | 0.74 | 4.29E-05 | 0.000936 |
| hsa-miR-5010-3p | 0.74 | 5.60E-05 | 0.001177 |
| hsa-miR-101-1-3p | -1.49 | 6.88E-05 | 0.001354 |
| hsa-miR-550a-1-5p | 0.62 | 6.93E-05 | 0.001354 |
| hsa-let-7a-1-5p | -0.69 | 7.66E-05 | 0.001442 |
| hsa-miR-28-3p | 0.67 | 7.88E-05 | 0.001442 |
| hsa-miR-3157-3p | 0.89 | 8.79E-05 | 0.001558 |
| hsa-miR-1260b-5p | 2.12 | 9.96E-05 | 0.001712 |
| hsa-miR-126-3p | -1.14 | 0.000107 | 0.001778 |
| hsa-miR-6505-3p | 1.40 | 0.00011 | 0.001778 |
| hsa-miR-190a-5p | -1.29 | 0.000116 | 0.001827 |
| hsa-miR-29b-1-3p | -0.81 | 0.000124 | 0.001894 |
| hsa-miR-26b-5p | -0.94 | 0.000133 | 0.001953 |
| hsa-miR-548k-5p | -1.07 | 0.000134 | 0.001953 |
| hsa-miR-30c-1-5p | 0.72 | 0.000145 | 0.00206 |
| hsa-miR-7846-3p | -1.18 | 0.000163 | 0.002255 |
| miRNA | logFC | PValue | FDR |
| hsa-miR-5703-3p | 3.16 | 0.000207 | 0.002801 |
| hsa-let-7d-3p | 0.90 | 0.000224 | 0.002953 |
| hsa-miR-3127-3p | 0.97 | 0.000242 | 0.003114 |
| hsa-miR-3182-5p | 1.28 | 0.000269 | 0.003385 |
| hsa-miR-6513-3p | 0.65 | 0.000296 | 0.003576 |
| hsa-miR-374a-5p | -1.33 | 0.000296 | 0.003576 |
| hsa-miR-6765-3p | 0.98 | 0.000303 | 0.003579 |
| hsa-miR-628-3p | -0.90 | 0.000351 | 0.004064 |
| hsa-mir-6501 | 3.05 | 0.000373 | 0.00423 |
| hsa-miR-6762-3p | 1.07 | 0.00043 | 0.004785 |
| hsa-miR-2861-3p | -1.11 | 0.000577 | 0.006296 |
| hsa-miR-4780-3p | 2.85 | 0.00061 | 0.00653 |
| hsa-miR-598-3p | 2.38 | 0.000647 | 0.006772 |
| hsa-let-7b-3p | 0.61 | 0.000657 | 0.006772 |
| hsa-miR-3614-3p | 2.49 | 0.000739 | 0.00748 |
| hsa-miR-126-5p | -1.21 | 0.000809 | 0.008048 |
| hsa-miR-6125-3p | -1.09 | 0.000837 | 0.00818 |
| hsa-miR-6837-3p | 0.92 | 0.00088 | 0.008354 |
| hsa-mir-4422 | 1.84 | 0.000888 | 0.008354 |
| hsa-miR-186-5p | 0.71 | 0.000899 | 0.008354 |
| hsa-miR-548e-3p | -1.12 | 0.001104 | 0.010098 |
| hsa-miR-1273c-3p | 0.68 | 0.001186 | 0.010674 |
| hsa-miR-106b-3p | -0.53 | 0.001362 | 0.012067 |
| hsa-miR-4659a-3p | 0.76 | 0.001524 | 0.012968 |
| hsa-miR-328-3p | 0.61 | 0.00153 | 0.012968 |
| hsa-miR-4742-3p | 0.62 | 0.001547 | 0.012968 |
| hsa-miR-4435-1-5p | 0.62 | 0.001555 | 0.012968 |
| hsa-miR-532-5p | -0.68 | 0.001866 | 0.015338 |
| hsa-miR-330-5p | 0.62 | 0.001917 | 0.015529 |
| hsa-miR-6842-3p | 0.57 | 0.002077 | 0.016474 |
| hsa-mir-6867 | 2.86 | 0.002092 | 0.016474 |
| hsa-miR-3909-3p | 0.67 | 0.002187 | 0.016813 |
| hsa-miR-625-5p | 0.68 | 0.002194 | 0.016813 |
| hsa-mir-922 | 2.33 | 0.002235 | 0.016894 |
| hsa-mir-1273a | 0.46 | 0.002269 | 0.016925 |
| hsa-miR-5690-5p | 0.77 | 0.002412 | 0.017608 |
| hsa-mir-144 | 1.71 | 0.002422 | 0.017608 |
| hsa-miR-338-3p | -0.83 | 0.002467 | 0.017704 |
| hsa-miR-4661-5p | 1.11 | 0.002559 | 0.018138 |
| hsa-miR-425-5p | 0.53 | 0.002996 | 0.020975 |
| hsa-miR-3168-5p | 1.72 | 0.003049 | 0.02098 |
| hsa-miR-3074-5p | 0.47 | 0.003088 | 0.02098 |
| hsa-miR-1301-3p | -0.70 | 0.003111 | 0.02098 |
| hsa-miR-16-2-3p | -0.72 | 0.003181 | 0.02098 |
| hsa-mir-212 | -1.24 | 0.003182 | 0.02098 |
| miRNA | logFC | PValue | FDR |
| hsa-miR-340-3p | 0.72 | 0.0033 | 0.021505 |
| hsa-miR-30d-5p | 0.54 | 0.003379 | 0.021769 |
| hsa-miR-7155-3p | 0.96 | 0.003454 | 0.022003 |
| hsa-mir-7846 | -0.94 | 0.003524 | 0.022202 |
| hsa-miR-6859-1-3p | 0.76 | 0.00359 | 0.02237 |
| hsa-mir-4501 | 2.19 | 0.003703 | 0.022821 |
| hsa-miR-1285-1-5p | 0.53 | 0.003985 | 0.024296 |
| hsa-miR-5695-3p | -0.61 | 0.004071 | 0.024558 |
| hsa-miR-6754-3p | 0.91 | 0.004293 | 0.025624 |
| hsa-miR-5701-1-5p | 1.03 | 0.004478 | 0.026448 |
| hsa-miR-5006-3p | 0.91 | 0.004543 | 0.026554 |
| hsa-miR-144-3p | -1.37 | 0.004664 | 0.026983 |
| hsa-miR-128-1-3p | 0.39 | 0.005081 | 0.029099 |
| hsa-miR-2110-5p | 0.52 | 0.005166 | 0.029293 |
| hsa-miR-1234-3p | -0.76 | 0.005232 | 0.029374 |
| hsa-miR-658-3p | -0.96 | 0.005332 | 0.02964 |
| hsa-miR-451a-3p | 0.76 | 0.005511 | 0.030337 |
| hsa-miR-484-5p | 0.52 | 0.005642 | 0.030351 |
| hsa-miR-6087-3p | -0.75 | 0.005663 | 0.030351 |
| hsa-miR-143-3p | -0.79 | 0.005674 | 0.030351 |
| hsa-miR-21-5p | -0.87 | 0.005969 | 0.031632 |
| hsa-miR-215-5p | 1.28 | 0.006105 | 0.032049 |
| hsa-miR-652-3p | -0.43 | 0.006263 | 0.032581 |
| hsa-mir-3648-1 | -0.63 | 0.006511 | 0.033563 |
| hsa-miR-19a-3p | -0.96 | 0.006571 | 0.033567 |
| hsa-miR-6866-3p | 0.95 | 0.006853 | 0.034676 |
| hsa-miR-371b-5p | 0.71 | 0.006911 | 0.034676 |
| hsa-miR-6820-3p | 0.54 | 0.007099 | 0.03521 |
| hsa-miR-3150a-3p | 0.69 | 0.007141 | 0.03521 |
| hsa-miR-191-5p | 0.56 | 0.007259 | 0.035252 |
| hsa-let-7i-5p | -0.74 | 0.007274 | 0.035252 |
| hsa-miR-223-3p | -0.60 | 0.007649 | 0.036756 |
| hsa-miR-6730-3p | -1.01 | 0.00791 | 0.03749 |
| hsa-miR-339-3p | 0.46 | 0.007934 | 0.03749 |
| hsa-miR-4779-5p | 0.75 | 0.008429 | 0.039499 |
| hsa-miR-589-5p | 0.40 | 0.008807 | 0.04093 |
| hsa-miR-92a-2-3p | 0.56 | 0.008924 | 0.041136 |
| hsa-miR-5581-3p | 0.76 | 0.009214 | 0.042134 |
| hsa-miR-29a-3p | -0.45 | 0.009587 | 0.043486 |
| hsa-miR-10a-5p | 0.60 | 0.01011 | 0.044754 |
| hsa-miR-301a-3p | -0.92 | 0.010156 | 0.044754 |
| hsa-miR-378a-5p | 0.57 | 0.01017 | 0.044754 |
| hsa-mir-4419b | 0.66 | 0.010182 | 0.044754 |
| hsa-miR-185-3p | -0.43 | 0.010442 | 0.045543 |
| hsa-let-7d-5p | -0.41 | 0.010579 | 0.045789 |
| miRNA | logFC | PValue | FDR |
| hsa-miR-2110-3p | 0.60 | 0.010673 | 0.045846 |
| hsa-miR-3150b-3p | -0.73 | 0.010981 | 0.046816 |
| hsa-miR-941-1-3p | -0.42 | 0.011314 | 0.047872 |
| hsa-mir-6087 | -0.91 | 0.011907 | 0.05001 |
| hsa-miR-148b-3p | -0.70 | 0.012002 | 0.050039 |
| hsa-miR-548e-5p | -0.57 | 0.012229 | 0.05061 |
| hsa-miR-16-1-5p | -0.65 | 0.012567 | 0.051633 |
| hsa-miR-103a-1-3p | -0.56 | 0.012684 | 0.051739 |
| hsa-miR-3605-3p | 0.63 | 0.012899 | 0.052241 |
| hsa-miR-197-3p | -0.43 | 0.013477 | 0.053868 |
| hsa-mir-1180 | -0.83 | 0.013491 | 0.053868 |
| hsa-miR-331-5p | -0.63 | 0.013604 | 0.05394 |
| hsa-miR-324-3p | -0.40 | 0.015007 | 0.05909 |
| hsa-miR-942-5p | 0.47 | 0.015473 | 0.060503 |
| hsa-miR-660-3p | -0.70 | 0.016348 | 0.06349 |
| hsa-miR-361-3p | -0.34 | 0.017253 | 0.066547 |
| hsa-miR-6818-3p | 0.65 | 0.018013 | 0.069011 |
| hsa-miR-4746-5p | 0.51 | 0.018308 | 0.069667 |
| hsa-miR-6806-3p | 0.72 | 0.018592 | 0.070279 |
| hsa-mir-3960 | -0.67 | 0.019282 | 0.072404 |
| hsa-miR-641-5p | 0.56 | 0.019789 | 0.073818 |
| hsa-miR-6735-3p | -0.79 | 0.020015 | 0.074172 |
| hsa-mir-4419a | -0.80 | 0.020824 | 0.076671 |
| hsa-miR-584-3p | 0.92 | 0.021199 | 0.077536 |
| hsa-miR-4531-3p | -0.74 | 0.021333 | 0.077536 |
| hsa-miR-4466-5p | -0.64 | 0.022704 | 0.081995 |
| hsa-miR-363-3p | 0.39 | 0.023963 | 0.085785 |
| hsa-mir-4259 | -0.76 | 0.024056 | 0.085785 |
| hsa-miR-6783-3p | 0.61 | 0.024479 | 0.086747 |
| hsa-miR-4508-5p | -0.77 | 0.026577 | 0.093331 |
| hsa-miR-502-3p | -0.63 | 0.026666 | 0.093331 |
| hsa-miR-4649-3p | 0.70 | 0.027738 | 0.096487 |
| hsa-miR-943-3p | 0.70 | 0.02896 | 0.100123 |
| hsa-miR-4472-2-3p | 0.41 | 0.029211 | 0.100379 |
| hsa-miR-15b-3p | -0.40 | 0.03053 | 0.104281 |
| hsa-miR-15b-5p | -0.35 | 0.031517 | 0.107006 |
| hsa-miR-3656-3p | -0.61 | 0.034123 | 0.114834 |
| hsa-miR-4753-5p | -0.74 | 0.034227 | 0.114834 |
| hsa-miR-1233-1-3p | 0.72 | 0.034573 | 0.11531 |
| hsa-miR-345-5p | 0.35 | 0.035022 | 0.115729 |
| hsa-miR-96-5p | -0.89 | 0.035106 | 0.115729 |
| hsa-miR-30e-3p | 0.40 | 0.03712 | 0.121659 |
| hsa-miR-4755-5p | 0.46 | 0.037441 | 0.122004 |
| hsa-miR-20a-5p | -0.74 | 0.038896 | 0.125545 |
| hsa-miR-607-5p | -0.46 | 0.03897 | 0.125545 |
| miRNA | logFC | PValue | FDR |
| hsa-miR-146b-5p | -0.35 | 0.039818 | 0.126947 |
| hsa-miR-3615-3p | 0.42 | 0.039853 | 0.126947 |
| hsa-miR-142-5p | -0.44 | 0.0402 | 0.127338 |
| hsa-miR-92b-3p | 0.49 | 0.040724 | 0.128279 |
| hsa-miR-3180-1-5p | 0.80 | 0.041344 | 0.129515 |
| hsa-miR-4419a-5p | -0.66 | 0.041787 | 0.130183 |
| hsa-miR-340-5p | -0.65 | 0.042029 | 0.130221 |
| hsa-mir-1254-1 | -0.82 | 0.043693 | 0.134642 |
| hsa-miR-7855-5p | 0.60 | 0.044835 | 0.137413 |
| hsa-miR-107-3p | -0.52 | 0.045441 | 0.138521 |
| hsa-miR-423-3p | 0.37 | 0.046655 | 0.141461 |
| hsa-miR-6819-3p | -0.67 | 0.048486 | 0.146232 |
| hsa-miR-4454-5p | 0.43 | 0.049364 | 0.148091 |
| hsa-miR-210-3p | -0.61 | 0.049764 | 0.148193 |
| hsa-miR-3173-5p | 0.58 | 0.04992 | 0.148193 |
